# Barriers, Enablers and Strategies for the Treatment and Control of Hypertension in Nepal: A Systematic Review

**DOI:** 10.3389/fcvm.2021.716080

**Published:** 2021-10-11

**Authors:** Raja Ram Dhungana, Zeljko Pedisic, Achyut Raj Pandey, Nipun Shrestha, Maximilian de Courten

**Affiliations:** ^1^Institute for Health and Sport, Victoria University, Melbourne, VIC, Australia; ^2^Nepal Health Research Council, Kathmandu, Nepal; ^3^Department of Primary Care and Mental Health, University of Liverpool, Liverpool, United Kingdom; ^4^Mitchell Institute for Education and Health Policy, Victoria University, Melbourne, VIC, Australia

**Keywords:** barriers, facilitators, hypertension, treatment, control, Nepal

## Abstract

**Background:** Understanding country-specific factors influencing hypertension care is critical to address the gaps in the management of hypertension. However, no systematic investigation of factors influencing hypertension treatment and control in Nepal is available. This study aimed to systematically review the published literature and synthesise the findings on barriers, enablers, and strategies for hypertension treatment and control in Nepal.

**Methods:** Embase, PubMed, Web of Science, CINAHL, ProQuest and WorldCat, and Nepali journals and government websites were searched for qualitative, quantitative, and mixed-methods studies on factors or strategies related to hypertension treatment and control in Nepal. Information from qualitative studies was analysed using template analysis, while results from quantitative studies were narratively synthesised. Summary findings were framed under “health system”, “provider”, and “patient” domains. The protocol was registered in PROSPERO (registration number: CRD42020145823).

**Results:** We identified 15 studies; ten related to barriers and enablers and five to strategies. The identified barriers associated with the health system were: lack of affordable services and lack of resources. The barriers at the provider's level were: communication gaps, inadequate counselling, long waiting hours for appointments, lack of national guidelines for hypertension treatment, and provider's unsupportive behaviours. Non-adherence to medication, irregular follow-up visits, lack of awareness on blood pressure target, poor help-seeking behaviours, reluctance to change behaviours, perceived side-effects of anti-hypertensive medication, self-medication, lack of family support, financial hardship, lack of awareness on blood pressure complications, and comorbidity were barriers identified at patient level. The following enablers were identified: free essential health care services, family support, positive illness perception, and drug reminders. Strategies implemented at the health system, provider and patient levels were: establishing digital health records at health centres, health worker's capacity development, and health education.

**Conclusion:** There is a range of barriers for hypertension treatment and control in Nepal pertaining to the health system, health providers, and patients. Comprehensive interventions are needed at all three levels to further improve management and control of hypertension in Nepal.

## Background

A political declaration on the prevention and control of non-communicable diseases (NCDs), adopted by the General Assembly of the United Nations in 2011, emphasises the importance of developing national capacities to deal with NCDs, particularly in low- and middle-income countries (LMICs) (1). Though more and more focus of the health system is placed on tackling NCDs, the rate of hypertension treatment and control in LMICs is low (2). Compared to high-income countries, LMICs reported substantially lower proportions of hypertension treatment (55.6 vs. 29.0%) and control (28.4 vs. 7.7%) in 2010 (3). In three South Asian countries, namely, Bangladesh, India, and Pakistan, 31.9% of the hypertensive population was on anti-hypertensive medication and only 12.9% had controlled blood pressure (4).

Nepal, a South Asian lower-middle-income country landlocked between China and India hosts a population of 28 million, with 125 caste/ethnic groups living mostly in rural areas (5, 6). Under the Ministry of Health and Population, the public health system in Nepal delivers health services to the community through the tertiary, district, and primary care centres and health posts. Alongside public health services, private for-profit sectors and non-governmental organisations provide health services in Nepal (7).

With the growing burden of hypertension, untreated and uncontrolled hypertension is prevalent in Nepal (8–12). The majority of hypertensive individuals were not aware of their condition (awareness = 40.0%); a half of those that were aware were not treated (treatment = 20.2%), and a half of those on treatment, the blood pressure was not controlled (control = 10.5%) in 2016 (13).

Gaps in hypertension treatment and control can be due to various factors associated with the health system, health care providers, and individual patients. Systematic reviews and other studies from outside of Nepal suggest that lack of access, poor service delivery, unaffordability of health services, and limited resources are common health system-related barriers (14–19). Similarly, studies also found that the factors pertaining to patients and providers, such as lack of awareness and treatment, non-adherence to medicine, adverse drug effect, therapeutic inertia, and communication gaps, were contributing to uncontrolled hypertension (16, 19–29).

The quantity and the scope of primary research on barriers, enablers, and strategies for hypertension control are growing in Nepal. While new evidence from primary studies is evolving, a systematic review is needed for a comprehensive identification, understanding and synthesis of the factors affecting hypertension treatment and control in the Nepali context. Therefore, this review aimed to systematically synthesise evidence on barriers, enablers, and strategies for hypertension treatment and control in Nepal. By providing a comprehensive understanding of health system- and individual-level barriers and enablers and strategies of hypertension treatment and control, this review aims to inform the policymakers and related stakeholders about the gaps in hypertension care and contribute to the development of the contextual and problem-specific strategies to increase hypertension control in Nepal.

## Theoretical Framework

**Figure 1:**
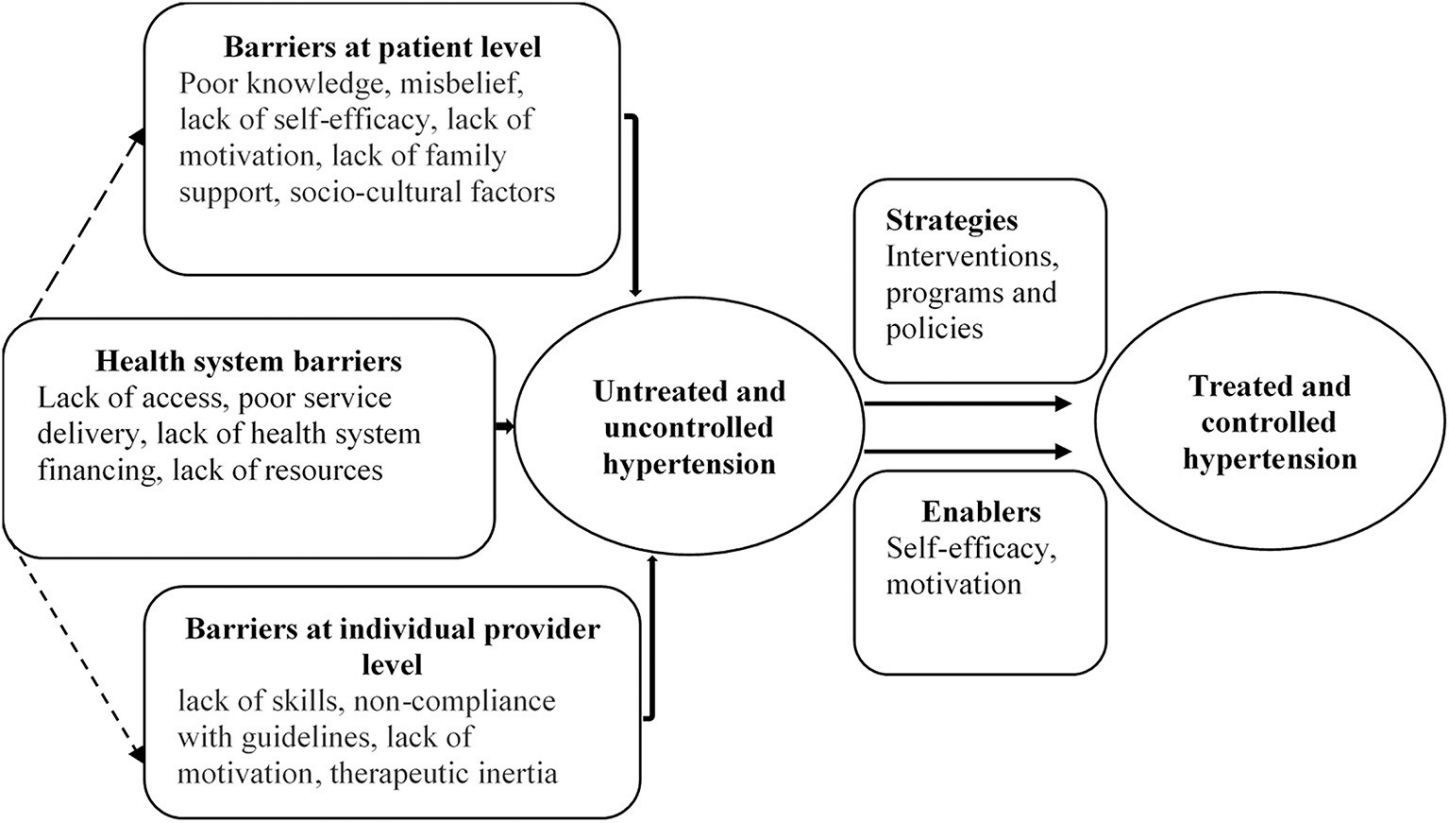
Conceptual framework of barriers, enablers, and strategies for hypertension control.

A framework was used to organise patient, health care provider, and health system (excluding patients and providers) barriers, enablers, and strategies for improving blood pressure control. The framework was derived from the theories used in previous studies on barriers (17, 19, 30), and adapted to also fit the enablers and strategies (Figure 1).

By “barriers”, we considered factors that prevent hypertension treatment and adequate blood pressure control (30). These have previously been linked with hypertensive patients, providers, and health system (30). Patient-reported barriers include, for example, knowledge, perception, beliefs, practises, self-efficacy, motivation, family support, affordability, and socio-cultural factors such as social dining and drinking (19, 31). These factors may affect health-seeking behaviours, treatment adherence, and follow-up care among patients, ultimately affecting blood pressure control (30). The commonly cited barriers by the providers comprise a lack of skills, non-compliance with guidelines, lack of motivation, and therapeutic inertia (19, 29, 30). An interaction of patient and provider factors can sometimes lead to a communication gap and a poor relationship, affecting treatment adherence and follow-up care (32).

Furthermore, enablers are the personal or contextual factors that facilitate actions or a required behaviour for hypertension treatment and control. They include, for example, perceived severity of illness and family support (33).

Strategies to improve blood pressure control include interventions, programs, or policies targeted at the providers, patients, or other components of the health system. Some of the proposed evidence-based strategies are: task shifting (34); team-based care (35); health system financing, including insurance coverage and co-payments of medical care (17); health system arrangement, including improved service delivery (36); using combination therapy (37) or polypill (38); home-based blood pressure monitoring (39); and educational interventions for patients and physicians (40).

## Methods

We systematically searched the literature, selected eligible studies, assessed methodological quality, extracted relevant data and synthesised findings in accordance with the PRISMA (Supplementary Table 1) (41) and Synthesis Without Meta-Analysis (SWiM) guidelines (Supplementary Table 2) (42). The protocol has been registered in PROSPERO (registration number: CRD42020145823).

### Eligibility Criteria

We used the PICOS (P-Population; I- Intervention/Exposure; C-Control/Comparator; O-Outcome; S- Study design) model to frame our research questions and applied the same model to define the eligibility criteria for study selection.

#### Population

We included studies that provided information on any factors limiting or facilitating the performance of hypertension treatment and control in Nepal. These factors were associated with hypertensive patients, health care providers (physicians, nurses, health care workers, and others), or health system. We did not restrict studies according to participants' age, gender, ethnicity, or comorbidity.

#### Exposure/Intervention

We defined the exposure as any factors that reportedly impede or facilitate hypertension treatment and control in Nepal (17, 30), excluding the non-modifiable personal attributes such as age, gender and ethnicity of patients and providers. Strategies were any interventions, program or policies targeted to the providers or patients or other components of health system and delivered at the community or health facility level for improving hypertension treatment and control.

#### Comparator/Control

We included studies with or without comparators or control groups. Some of the comparators or controls were untreated, uncontrolled hypertensive individuals, general population, or patients who receive usual care.

#### Outcomes

The outcomes of interest were hypertension treatment, hypertension control, and treatment adherence. By “hypertension treatment” we considered the use of at least one antihypertensive medication by hypertensive patients (17). We defined “hypertension control” as maintaining systolic blood pressure of <140 mmHg and diastolic blood pressure of <90 mmHg in individuals under antihypertensive medication (17). “Medication adherence” was defined as consistently taking antihypertensive medication as prescribed by the health care provider (17).

#### Types of Study

We included observational (qualitative, quantitative, and mixed-methods) and experimental studies that assessed and quantified barriers, enablers, and strategies for hypertension treatment and control in Nepal. Qualitative studies that provided information on views and experiences of patients, providers, and other related stakeholders regarding barriers and enablers of hypertension treatment and control were also included in the review. Studies were included regardless of language of publication or sample size. Reviews, case reports, case series, and conference abstracts were not deemed eligible for inclusion.

### Search Strategy

The primary literature search was conducted in PubMed, Embase, Web of Science, CINAHL through EBSCOHost, ProQuest, and WorldCat databases. We used a comprehensive search strategy to identify hypertension-related literature published between 2000 and 2020 in Nepal. Barriers, enablers, and strategies for hypertension treatment and control are likely to change over time. Therefore, to provide the most relevant data for informing changes in health system, this review focused on the current situation, and we only searched for the recent literature (i.e., published in the last 20 years). We combined the search terms “hypertension” and “blood pressure” with the search term “Nepal”. The full search syntax is provided in Supplementary Table 3.

The secondary search was done through Nepal Journals Online, Journal of Nepal Health Research Council, Journal of Kathmandu Medical College, Nepal Medical College Journal, Journal of Nepal Medical Association, and Chitwan Medical College Journal to identify any relevant citations we may have missed in the primary search. We also accessed websites of the Nepali Government, professional and regulatory organisation, and national and international agencies. Finally, we conducted forward citation searching for included studies using Google Scholar database and screened the reference lists of the included studies, as part of a backward citation search.

### Data Management

We used EndNote X8 (Clarivate Analytics, Philadelphia, PA, USA) and Rayyan QCRI (43) to manage the records and other data throughout the review. At first, we exported the citations to EndNote X8 to identify and remove the duplicates. The citations were then imported to Rayyan QCRI, to carry out screening and data extraction.

### Selection Process

**Figure 2:**
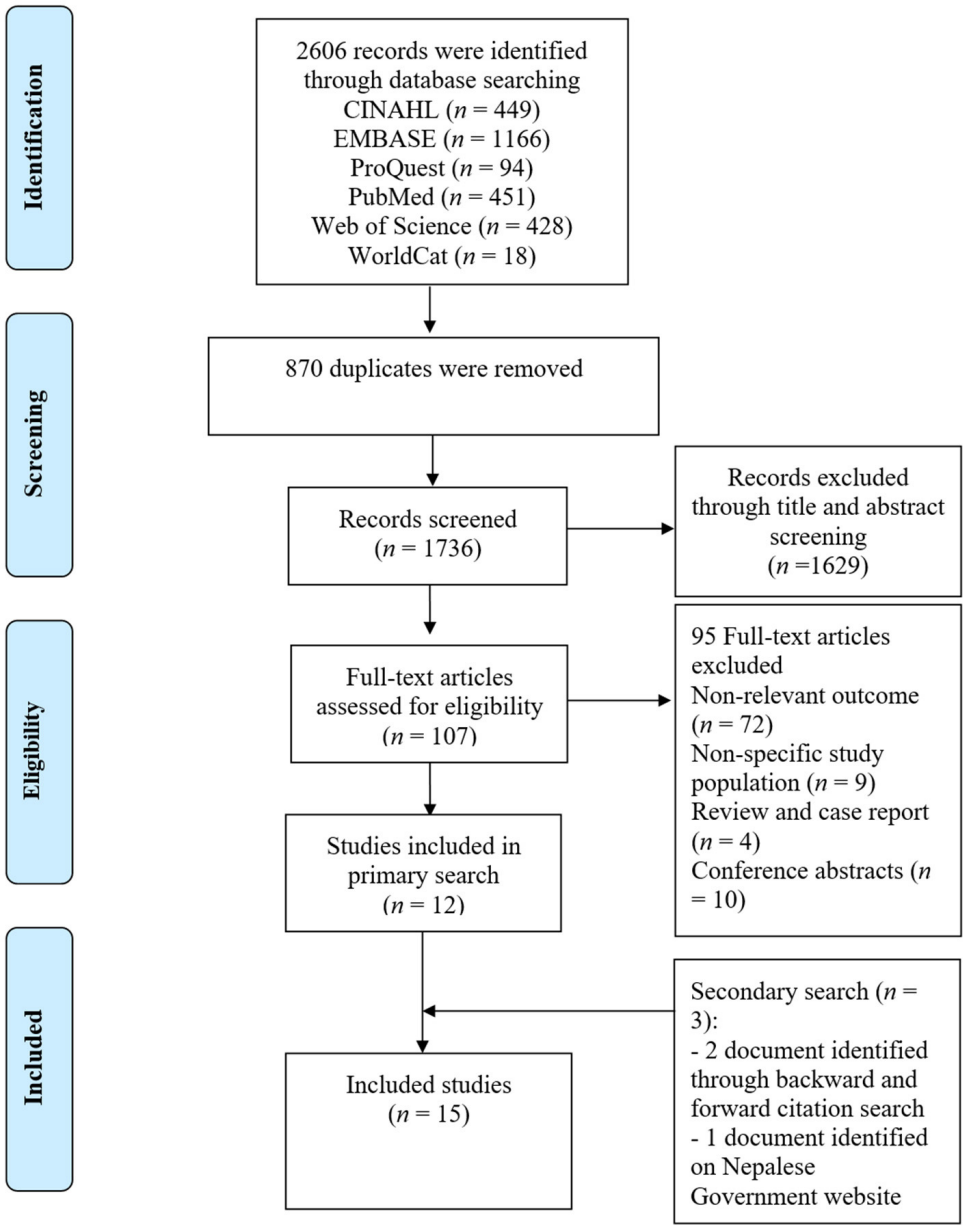
Study selection flow diagram.

Initially, two authors (RRD and ARP) independently screened the titles and abstracts of the articles and labelled them as “included”, “maybe”, and “excluded”. Full-texts of all records except the ones that were labelled “excluded” by both reviewers were reviewed. The screening process was blinded, and all disagreements in study selection were settled by consensus between the two authors. A PRISMA flow chart illustrating the study selection process is presented in Figure 2.

### Data Extraction

Using a pre-designed form, two authors (RRD and ARP) independently extracted the following data from the included publications: study design; study types; study participants; study setting; statistical method; year of publication; information to assess the risk of bias; types and/or prevalence of barriers, enablers, and strategies; and the effects on hypertension awareness, treatment, and control. For strategies, we additionally extracted data related to the comparator group.

### Quality Assessment

We used the Mixed Methods Appraisal Tool (MMAT) to assess the methodological quality of the selected studies. MMAT includes a five-item checklist for different types of study design, with “Yes” “No”, and “Can't tell” response options (Supplementary Table 4). For each “Yes” response, the given study was assigned a star (^*^). A study could, therefore, get an overall score between zero and five stars. For the purpose of this review, we used the following scoring system for overall methodological quality: 0–1 star = “low”; 2–3 stars = “fair”; 4–5 stars = “good”. Two authors (RRD and ARP) independently checked the quality of the studies. To resolve discrepancies between assessment scores provided by the two authors, a third author (NS) also assessed the quality of the studies using the same tools. All conflicts were resolved by consensus. We did not exclude studies from the review based on the outcome of quality assessment.

### Data Synthesis

Both qualitative and quantitative findings were discussed together to enrich the understanding of barriers and enablers of hypertension and control specific to health system, health care providers, and patients in the Nepalese context. To enable such a mixed-methods approach to synthesise the evidence, we incorporated both qualitative and quantitative data within the domains of previously described theoretical framework. This allowed us to provide a joint summary of both quantitative and qualitative findings from individual studies for each of the domains.

Extracted qualitative data were analysed using template analysis (44). Two authors of the current study used the pre-defined template to extract the meta code/code and associated text from the primary qualitative study and enable merging them into three major domains. The list and definition of the prior codes used in template analysis are given in Supplementary Table 5. The final list of codes was further categorised as “health system-related”, “Provider-related” and “Patient-related” barriers and enablers. The code relating to the institution, resource, finance and services were categorised as health system-related factors. Attitudes, perceptions, practises, behaviours, and beliefs of patients and providers were listed under their respective domains.

We calculated the odds ratios and their 95% confidence interval (CI) using the proportions of exposure and outcomes, where available. Due to the limited information on the exposures and outcome and large methodological heterogeneity between the selected studies, we did not conduct a meta-analysis. Instead, in attempt to identify the most prominent factors, we applied the vote counting method to count the frequency of their mentions across the studies, as suggested in the Cochrane Handbook for Systematic Reviews of Interventions (45). Before that, the factors for which ≥60% of studies showed a positive or negative association were assigned to the domains of health system, providers, and patients, in accordance with the framework.

## Results

### Study Selection

The systematic search of six electronic databases resulted in a total of 2,606 records. We removed 870 duplicates in Endnote and exported the remaining 1,736 records to Rayyan QCRI for the title and abstract screening. We excluded 1,629 ineligible titles and abstracts and retrieved the full-texts of the remaining 107. Out of 107 full-text articles that we assessed, 95 had either ineligible outcomes such as pulmonary hypertension, were conducted among non-hypertensive or non-Nepali participants, or were reviews, case reports or conference abstracts. We did the forward and backward citation tracking for the remaining 12 studies and identified two additional records (46, 47). One additional study eligible report was identified on the Nepalese Government website. No additional eligible studies were found in Nepalese journals and on websites of Nepali professional and regulatory organisations, and national and international agencies. Finally, we included 15 studies (32, 46–59) for qualitative synthesis (Figure 2). None of the studies were included in the quantitative synthesis of effect sizes because of the significant differences in their aims, methods, interventions/exposures, and outcome measures.

### Study Characteristics

The included studies were conducted in Bagmati province (Kathmandu and its periphery, *n* = 8), Gandaki province (Kaski, *n* = 4), province 1 (Sunsari, *n* = 2), and Sudurpaschim province (Achham, *n* = 1). The numbers of studies conducted in hospital (*n* = 8) and community (*n* = 7) settings were nearly equal. All the community-based studies included participants from the peri-urban (*n* = 3) and urban (*n* = 3) areas. Only a study conducted in Acham (hospital and community-based) included participants from a rural area (50). More than half of the studies were completed in 2015 or later. Ten studies assessed the barriers and enablers of hypertension treatment and control (32, 46, 47, 53–59). Of these, three studies applied qualitative methods (32, 54, 55), six used quantitative methods (46, 47, 53, 57–59), and one was conducted using mixed methods (56). The majority of these studies investigated hypertension treatment as an outcome of interest (Supplementary Table 6). Three studies also discussed adherence to antihypertensive medications (47, 57, 58). Most studies collected data from hypertensive participants, except one study that interviewed community health workers to explore the barriers and enablers in utilising health care among the patients (55).

Five of the included studies that assessed the effectiveness of hypertension treatment and control strategies (48–52) were prospective comparative studies (*n* = 1) (52), randomised trials (*n* = 2) (49, 51), and uncontrolled before and after studies (*n* = 2) (48, 50). Four out of these five studies reported systolic and diastolic blood pressure as outcome measures (48, 49, 51, 52). The studies tested the effectiveness of health education (48–52) and combined, antihypertensive medication, and yoga (51) interventions (Supplementary Table 7).

The total number of participants in the included studies was 3,279, with the individual sample sizes ranging from *n* = 13 to *n* = 1,638. Only three studies applied multivariable analysis and reported adjusted effect sizes (49, 57, 58). Most of the qualitative and mixed methods studies (three out of four) applied a thematic analysis. Three studies (32, 49, 50) reported non-response rates, and they ranged between 9 and 27.8%.

### Study Quality

In quality assessment, all studies received three or more stars, suggesting they were of fair or good methodological quality (Supplementary Table 4). Only three out of 11 quantitative studies (49, 57, 58) adjusted for confounding.

### Barriers and Enablers of Hypertension Treatment and Control

#### Barriers to Hypertension Treatment and Control

We identified several barriers to hypertension treatment and control discussed in both qualitative and quantitative studies. They are tabulated separately in Supplementary Tables 8, 9. We categorised all the barriers into three major domains: health system-related barriers, provider-related barriers, and patient-related barriers (Table 1).

**Table 1:** Barriers to hypertension treatment and control in Nepal.

**Domain**	**Barriers (category)**	**Barriers identified in**		**Study count**
		**Qualitative study**	**Quantitative study**	
Health system-related	Unaffordability of services and medicines	Unaffordable services (32)	Expensive (vs. affordable) medicine (57), high cost of medicine (59)	+++
	Lack of human resources, diagnostic tools and medicines	Lack of human resources and diagnostic tools (55)	Unavailability of medicine (59)	++
Health care provider-related	Communication gap between patients and providers regarding medication use and follow-up visits	Communication gap between patients and providers regarding medication use and follow-up visits (32, 54–56)	–	++++
	Inadequate counselling on lifestyle modifications	Inadequate counselling on lifestyle modifications (32, 54–56)	No counselling (56)	++++
	Long waiting hours	Long waiting hours (32, 56)	long waiting hours (>20 min vs. ≤ 20 min) (56)	++
	Lack of national guidelines for hypertension treatment	Lack of national guidelines for hypertension treatment (56)	–	+
	Provider's unsupportive- behaviours	Provider's unsupportive- behaviours (32)		+
Patient-related	Non-adherence	Non-adherence (32, 56)	Poor adherence to medication (low adherence vs. high adherence) (56), Non-adherence to medication (53)	+++
	Irregular follow-up visits	Irregular follow-up visits (55, 56)	Lost to follow up (irregular vs. regular) (57), lost to follow up (no follow up vs. regular follow-up) (56), lack of blood pressure monitoring (53)	++++
	Lack of awareness on blood pressure target	–	Lack of awareness on blood pressure target (56), Not aware of normal blood pressure (53)	++
	Poor help-seeking behaviours	Poor help-seeking behaviours (55)	–	+
	Reluctance to change behaviours	Reluctance to change behaviours (32)	–	+
	Perceived side-effects of anti-hypertensive medication	Perceived side effects of anti-hypertensive medication (32, 56)	Perceived side effect of drugs (59)	+++
	Self-medication	Self-medication (55)	–	+
	Lack of family support	Lack of family support (32)	–	+
	Financial hardship	Financial hardship (54)	–	+
	Lack of awareness on blood pressure complications	–	lack of awareness on high blood pressure complications (53)	+
	Comorbidity	–	Comorbidity (yes vs. no) (58)	+

“+” *indicates the number of studies that reported the barriers*.

##### Health System-Related Barriers

The unaffordability of health services and medicines (32, 57, 59) and lack of human resources, diagnostic tools and medicines (55, 59) were the most often cited health system barriers.

##### Provider-Related Barriers

The most frequently cited barriers at providers' level were the communication gap between providers and patients (32, 54–56), health worker's lack of interest in counselling for lifestyle modifications (32, 54–56), and long waiting hours for the appointment (32, 56). Health care providers failed to deliver clear information to patients regarding medication dosage and duration, behaviour modification and need for routine monitoring. A hypertensive male participant from the study conducted by Shrestha et al. (32) complained that the doctor did not explain enough about his condition.

“….. *After check-up, I was told to take medicine. …..I was not told anything. So, I asked people who have heart disease to get the information regarding what food to eat, which food increases it, and which food controls pressure*” (32).

In addition, other provider-related factors affecting hypertension treatment and control were: lack of national guidelines in hypertension treatment (56) and provider's unsupportive behaviours (32).

##### Patient-Related Barriers

Both quantitative and quantitative studies reported various individual/patient-level factors that were significantly associated with untreated or uncontrolled hypertension. The most prominent barriers were: non-adherence to medication (32, 53, 56), irregular follow-ups (55–57), and patients' lack of awareness of the ‘normal’ blood pressure target (53, 56) (Table 1). For example, a 55 years old participant with uncontrolled hypertension from a study in Kathmandu reported not visiting the doctor for one and a half years since her last visit (56), as the doctor did not recommend regular visits.

“*I have not gone for follow-up. I am following the same regimen from the last one and a half years. My doctor told me to visit him only if I had problems*” (56).

Additionally, a wide variety of themes evolved from the qualitative analysis of the findings on patient's beliefs and practises that affect hypertension treatment and control (Supplementary Table 8). The factors impeding initiation of anti-hypertensive treatment were poor help-seeking behaviours (55) and reluctance to take medication due to perceived side effects and fear of long-term use (32, 56).

#### Enablers of Hypertension Treatment and Control

Three qualitative studies discussed facilitating factors for improved hypertension treatment. Among them, family support, positive illness perception, and using drug reminder were the most frequently cited ones (32, 54).

The remaining enablers were: provision of free essential medicine (55) and patient's motivation to control hypertension (32) (Table 2).

**Table 2:** Enablers and strategies for hypertension treatment and control in Nepal.

**Domain**	**Enablers**			**Strategies**
	**Category**	**Qualitative study**	**Quantitative study**	
Health system- related	Free essential medicines	Free essential medicines at health centre (55)		Strengthening data recording systems at the health care centre (50)
Health care provider-related				Capacity development of health worker (50, 52)
Patient- related	Family support	Family support (32, 54)		Health education for hypertensive patients (48–50, 52), yoga (51)
	Positive illness perception	Perceived seriousness of the illness	Positive illness perception (47)	
	Self-motivation	Self-motivation (32)		
	Drug reminder (packaging and text)	Use of medication containers (32)		
		Use of reminders for medication (32)		

Similarly, only one quantitative study discussed enablers separately. The study found that scores of dimensions of illness perception particularly timeline, treatment control, and coherence were positively correlated with medication adherence (47). The higher the participant perceived high blood pressure as a chronic condition (Spearman correlation coefficient (*r*) = 0.23, *p* < 0.05), the better the medication adherence was. The more the participant believed that the treatment can control the blood pressure (*r* = 0.51, *p* < 0.05), the higher the medication adherence score they had. The better the understanding of hypertension (*r* = 0.22, *p* < 0.05), the higher the medication adherence was (47).

### Strategies for Hypertension Treatment and Control

Out of five eligible studies in this category, all studies intervened at the patient level, two studies also investigated the effect of workforce strengthening (training and continuing education for integrated of NCD care), and one study evaluated the impact of new data recording systems at the health care centre (Table 2). The most frequently tested antihypertensive strategy was the health education for hypertensive patients (48–50, 52), and three out of four studies found it to be effective for reducing blood pressure (Table 3). Health education particularly related to lifestyle modification and medication reconciliations was applied as a component of comprehensive blood pressure management strategies (49, 50, 52) or as a single intervention (48). One study (52) also compared hydrochlorothiazide, enalapril, and amlodipine as first-line antihypertensive drugs. The reduction in mean systolic and diastolic pressure was significantly higher with enalapril and amlodipine, compared with hydrochlorothiazide (52). The participant under amlodipine reported more adverse events such as peripheral oedema, shortness of breath, and headache than the enalapril group.

**Table 3:** Findings of the studies on the effectiveness of strategies to improve hypertension treatment and control in Nepal.

**Study ID**	**Comparison**	**Sample size total (treatment/** **control)**	**Participants in analysis**	**Outcome**	**Findings**	**Overall MMAT score**
Humagain et al. (52)	Comparison 1: hydrochlorothiazide 25 mg vs. amlodipine 5 mg	172 (NA/NA)	172	Outcome1: Systolic blood pressure	Before and after difference of mean (sd) 14.6(5.1) mmHg vs. 21.9 (5.9) mmHg, *p* < 0.01	***
				Outcome 2: Diastolic blood pressure	Before and after difference of mean (sd) 8.8 (2.5) mmHg vs. 14.2 (2.8), *p* < 0.01	
	Comparison 2: hydrochlorothiazide 25 mg vs. enalapril 5 mg	172 (NA/NA)	172	Outcome 1: Systolic blood pressure	Before and after difference in mean (sd) 14.6 (5.1) mmHg vs. 21.8 (7.4), *p* < 0.01	
				Outcome 2: Diastolic blood pressure	Before and after difference in mean (sd) 8.8(2.5) mmHg vs. 14.2(2.9) mmHg, *p* < 0.01	
	Comparison 3: amlodipine 5 mg vs. enalapril 5 mg	172 (NA/NA)	172	Outcome 1: Systolic blood pressure	Before and after difference in mean (sd) 21.9 (5.9) mmHg vs. 21.7 (7.4) mmHg, *p* = 0.92	
				Outcome 2: Diastolic blood pressure	Before and after difference in mean (sd) 14.2 (2.8) mmHg vs. 14.3 (2.9), *p* = 0.86	
Khadka et al. (51)	yoga vs. routine care	14 (7/7)	14	Outcome 1: Systolic blood pressure	Before and after difference in median was 21 mmHg in yoga vs. 12 mmHg in control group, *p* < 0.05	***
				Outcome 2: Diastolic blood pressure	Before and after difference in median 18 mmHg in yoga group vs. 0 mmHg in control group, *p* < 0.05	
Kumar et al. (50)	multilevel intervention, no comparison group	340 (340/-)	340	Hypertension control	No statistically significant difference between baseline and endline outcome; hypertension control rate decreased from 75% (254 out of 340) to 73% (249 out of 340)	***
Neupane et al. (60)	Health education, blood pressure monitoring, and referral vs. usual care	1,638 (939/699)	1,468	Outcome 1: Systolic blood pressure	Before and after difference in mean was 6·47 mmHg in the intervention vs. 2·85 mmHg in the control group	****
				Outcome 2: Diastolic blood pressure	Before and after difference in mean was 2·90 mmHg in the intervention group vs. 1.11 mmHg in control group	
Sharma et al. (48)	Health education, no comparison group	50 (50/-)	50	Outcome 1: Systolic blood pressure	The mean (sd) reduced from 150.1 (7.8) mmHg to 137.7 (9.9 mmHg), *p* < 0.01	***
				Outcome 2: Diastolic blood pressure	The mean (sd) reduced from 104 (9.5) mmHg to 94.5 (7.7) mmHg, *p* < 0.01	

One study studied yoga as an adjuvant therapy to medication and compared it with usual care (51). The yoga group had a significant reduction in both systolic and diastolic blood pressure, compared with the “medication-only” group after 6 weeks of follow-up.

One of the two studies that intervened at the level of health care providers found a significant improvement in blood pressure amongst their clients. The study trained and involved female community health volunteers for health education, blood pressure monitoring, and referral of hypertensive cases. The intervention was effective in reducing blood pressure. The mixed-effect regression coefficient was −4.9 (95% CI: −7.8 to −2.0) for systolic blood pressure and −2.6 (95% CI: −4.6 to −0.7) for diastolic blood pressure (49). One study implemented the intervention program at health system level, by establishing a new digital and electronic health record system along with capacity development for health care workers and providing health education to the patients. The intervention had no statistically significant effect on hypertension control (50).

## Discussion

We systematically reviewed 15 studies to identify barriers, enablers, and strategies of hypertension treatment and control in Nepal. Ten of them investigated various barriers and enablers within three domains: the health system, health care providers, and patients. The remaining six studies investigated the effectiveness of different hypertension treatment and control strategies, including health education and yoga. Our study identified several barriers for hypertension treatment and control in Nepal. Most of them were related to health care providers and hypertensive patients such as communication gaps, inadequate counselling, long waiting hours for appointments, lack of national guidelines for hypertension treatment, provider's unsupportive behaviours, non-adherence to medication, irregular follow-up visits, lack of awareness on blood pressure target, poor help-seeking behaviours, reluctance to change behaviours, perceived side-effects of anti-hypertensive medication, self-medication, lack of family support, financial hardship, lack of awareness on blood pressure complications, and comorbidity. To improve hypertension treatment and control, these barriers need to be addressed in future interventions targeting hypertensive patients and health care providers.

The synthesis of the findings reported in qualitative studies generated two themes on health system-related barriers for hypertension control and treatment. They were the lack of affordable services and health system resources including human and physical assets. Barriers relating to the availability and affordability of hypertension care services are common in low-income countries (17, 19). The health system factors are complex and can have a strong influence on health service delivered by the provider and patient's access to care (17). The unaffordability of the services and financial hardship at the individual level were associated with poor help-seeking behaviours, non-adherence to medication and irregular follow-up visits to health centres among hypertensive patients. The quantitative studies that investigated barriers to hypertension treatment and control also showed a significant association between high cost of drugs and non-adherence to medication. The odds of not adhering to taking antihypertensive drugs were 5.2 times higher among the patients who reported the cost of medicine as expensive (57). This study identified the provision of free essential medicines at government health facilities as a single facilitating factor relating to the Nepali health system. Providing free essential antihypertensive medicines at the primary health care centres was supposed to increase access to treatment of hypertension. Some of the calcium channel blockers and beta blockers are freely available at primary health care centres. However, regular, effective, and efficient supply of these medicines is uncertain (61). For example, most of the health facilities (HF) reported unavailability of atenolol (82% of HF) and amlodipine (90% of HF) in 2015 (62).

None of the strategies from the selected studies aimed either to minimise the financial burden associated with hypertension treatment or to increase access to hypertension care. Only one study evaluated the effects of a strategy implemented at health system level. It developed and evaluated a new hospital-based health-records system, but the strategy failed to show a significant impact on blood pressure control. To increase access to care and minimise excessive health expenditures, different health system financing approaches, including health insurance coverage and co-payments for medication, have been evaluated globally (17). Nepal recently enacted the National Health Insurance Act 2017 and is moving towards universal health coverage for equitable access to high quality and affordable health care (63). However, its overall impact on treatment and control of hypertension and its related complications has yet to be studied.

The communication gap between health workers and patients was one of the most frequently studied barriers associated at provider level. The lack of clear messages on medication, dosage, lifestyle modification, and follow-up visits was reported as causes of non-adherence to medication and irregular follow-up visits, ultimately leading to suboptimal control of blood pressure. A recent study found that less medication (antihypertensive drugs) discussion between providers and patients was associated with six-fold increased odds of poor adherence to medication (64). Jolles et al. also emphasised that effective communication between providers and patients could increase medication adherence and contributes to better control of high blood pressure (65).

Capacity development of the female health community volunteers and involving them for health education, blood pressure monitoring and linking the cases to health services was the only effective intervention implemented at the provider level. involvement of female community health volunteers in screening the high-risk population and monitoring the hypertensive patients is cost-effective (60) and could minimise the burden of exhausted health care workers. After a very acclaimed performance on improving maternal and child health (66), new evidence suggests that community health workers could potentially work as the frontline cadres to fight against the burden of NCDs, including hypertension (67). Task sharing, particularly with other non-physician health workers in understaffed and resource-poor settings, is an effective strategy for the management of hypertension (34). Additionally, other approaches such as including pharmacists and nurses in a team and providing team-based hypertension care also showed positive effects on controlling high blood pressure (35).

Most of the barriers at the patient level were related to their knowledge, beliefs, and practises. Being unaware of the normal blood pressure target, perceived side effects of drugs, fear of long-term use of medication, reluctance to take medicine, self-medication, non-adherence to medication, lack of regular follow-up visits, lack of blood pressure monitoring, and not minimising risky health behaviours were the key barriers to hypertension treatment and control. Similar to our findings, Khatib et al. in their systematic review reported several patient-related barriers to hypertension treatment and control (19). The authors categorised them as capability barriers (e.g., lack of skills and knowledge), intention and motivation barriers (e.g., lack of motivation to adhere to a treatment, false belief, such as drug dependency), and medication barriers (e.g., self-perceived side effects, taste and dosage), among others (19). Interestingly, for most of the reported barriers they did not find large differences between LMICs and high-income countries.The only exception were availability barriers, such as lack of health care facilities and resources, which were more prominent in LMICs (19) as well as in Nepal.

Health education was the most commonly investigated strategy. One study used yoga as an adjuvant therapy to the medication and found that yoga was effective in blood pressure reduction. Globally, barriers relating to patient's knowledge, belief, and practises have been tackled effectively using different approaches, such as simplifying the treatment using polypill (38) and increasing adherence to medication, routinely and conveniently monitoring hypertension through home-based blood pressure measurement (39) and providing education interventions to patients and physicians (40). Future studies should evaluate the effectiveness of these strategies in the context of Nepali health system.

The current study provided a comprehensive insight into factors influencing hypertension treatment and control at the health system, health provider, and patient levels. The findings may be used to inform and guide relevant stakeholders while designing strategies for improving hypertension treatment and control in Nepal. Enabling access to essential antihypertensive medications at primary health care facilities and developing hypertension treatment protocols and guidelines for primary health care providers may be needed to improve curative care of hypertension in Nepal. Providing health education to hypertensive patients seems to be a viable strategy to improve hypertension care in Nepal. Increasing the involvement of female community health volunteers in the provision of health education may be helpful. Additionally, yoga could be recommended as a lifestyle therapy to manage hypertension as part of primary care. However, more research is needed to evaluate the effectiveness and feasibility of yoga interventions for hypertension treatment and control in the Nepalese context.

The use of information and communication technology-enabled integrated care has shown promising effects on blood pressure reduction in hypertensive patients (68, 69). Therefore, it may be worthwhile to explore the effectiveness and feasibility of telephone follow-ups, web-based and computer-tailored solutions, home-use devices for blood pressure monitoring, smartphone applications, and telemedicine in the Nepalese context. A recently published protocol announced a study that will aim to recruit 200 hypertensive patients in Nepal and evaluate feasibility of a mobile phone text messaging intervention (70).

A recent study indicated that there is a growing burden of NCD multimorbidity (i.e., occurrence of two or more chronic conditions) in Nepal (71). In response to this challenge, it would seem worthwile to evaluate integrated chronic care models for NCDs, including hypertension (50).

Some limitations of this review stem from the characteristics of the included studies. Although the majority of studies scored three out of five in MMAT checklists, nearly all of them were subject to inadequate reporting of research methods. For example, out of six comparative or intervention studies, three studies did not present clear information on the estimation of the required sample size and participant selection. Furthermore, only two quantitative studies adjusted their analyses for confounding. These methodological limitations of the included studies may have affected their findings and consequently the findings of our review.

A meta-analysis could not be performed due to a large methodological heterogeneity and variation in outcome measures between the included studies. For example, the data on barriers and enablers were extracted from qualitative, quantitative, and mixed methods studies. The data on antihypertensive strategies were from prospective comparative studies, randomised trials and uncontrolled before and after studies. The intervention modalities also varied across studies from health education to yoga.

## Conclusion

By reviewing 15 individual studies of fair-to-good methodological quality, we found a range of factors at the health system, health care provider, and patient levels that are likely to impede or facilitate hypertension treatment and control in Nepal. We also found that health education (provided by female community health volunteers and health workers) and yoga are promising interventions for hypertention treatment and control in Nepal. However, further studies are required to confirm their effectiveness and feasibility, before incorporating them in the clinical practise. Overall, the findings of our systematic review may assist policy makers and other public health stakeholders in designing future interventions to improve hypertension treatment and control in Nepal.

## Data Availability Statement

The original contributions presented in the study are included in the article/Supplementary Material, further inquiries can be directed to the corresponding author.

## Author Contributions

RD developed the study protocol, screened the records, extracted the data, assessed the quality of included studies, interpreted the findings, and prepared the first draft of the manuscript. AP screened the records, extracted the data, assessed the quality of included studies, and assisted in interpreting the findings. NS assisted in drafting and assessing the quality of the selected studies. ZP and MC conceptualised and reviewed the draft. All authors contributed to writing the manuscript and its revisions. All authors read and approved the final manuscript.

## Conflict of Interest

AP was employed by the company Abt Associates. The remaining authors declare that the research was conducted in the absence of any commercial or financial relationships that could be construed as a potential conflict of interest.

## Publisher's Note

All claims expressed in this article are solely those of the authors and do not necessarily represent those of their affiliated organizations, or those of the publisher, the editors and the reviewers. Any product that may be evaluated in this article, or claim that may be made by its manufacturer, is not guaranteed or endorsed by the publisher.
