# Supplementary material for: Barriers, Enablers and Strategies for the Treatment and Control of Hypertension in Nepal: A Systematic Review
Source: Front Cardiovasc Med. 2021 Oct 11;8:716080. doi: 10.3389/fcvm.2021.716080 (PMC8542767; doi:10.3389/fcvm.2021.716080)
Supplement: Supplementary file 3 [file Table_3.docx]

**Table 3. Search syntax**

| Sn | Search Engine | Syntax |
| --- | --- | --- |
| 1 | PubMed | (hypertension OR "blood pressure") AND Nepal* NOT animal |
| 2 | CINAHL | TX (hypertension OR “blood pressure”) AND TX Nepal NOT TX animals |
| 3 | Web of Science | ((hypertension OR "blood pressure")) AND TOPIC:(Nepal) NOT TOPIC: (Animal) |
| 4 | Embase | (hypertension OR “blood pressure”) AND Nepal NOT animal |
| 5 | ProQuest | (Hypertension OR "blood pressure") AND Nepal NOT Animal |
| 6 | WorldCat | (Hypertension OR "blood pressure") AND Nepal NOT Animal |
