# Supplementary material for: Barriers, Enablers and Strategies for the Treatment and Control of Hypertension in Nepal: A Systematic Review
Source: Front Cardiovasc Med. 2021 Oct 11;8:716080. doi: 10.3389/fcvm.2021.716080 (PMC8542767; doi:10.3389/fcvm.2021.716080)
Supplement: Supplementary file 6 [file Table_6.docx]

**Table 6. Characteristics of the studies on barriers and enablers of hypertension treatment and control in Nepal**

| **Sn** | **Study ID** | **Study types** | **Sampling method** | **Data collection method** | **Participants** | **Sample size total (m/f)** | **Survey year** | **Survey site** | **Area** | **Outcome** | **Comments** | **MMAT**  **scores** |
| --- | --- | --- | --- | --- | --- | --- | --- | --- | --- | --- | --- | --- |
| **1** | Bhandari et al. (2015) | Cross-sectional, quantitative study | Random sampling (154 from 975 hypertensive participants) | Semi-structured interview | Hypertensive persons of age 35 years and above | 154 (NA/NA) | September 2009 to February 2010 | Dharan sub-metropolitan city | Urban | Adherence to medication, hypertension control | Sex, education, occupation, family history of hypertension were adjusted. No information on gender distribution | **** |
| **2** | Devkota et al. (2016) | Cross-sectional, mixed-methods study | Stratified random sampling for the quantitative part | Structured interview | Hypertensive persons of age between 18 years and 70 years  - | 118 (54/64) | January to July 2015 | Kathmandu, Nepal | Peri-urban | Hypertension awareness, hypertension treatment, | No adjustment of confounding in analysis | *** |
|  |  |  | purposive  sampling in the qualitative component | Key informant interview | Cardiologist | 2 (2/0) | January to July 2015 | Kathmandu, Nepal | Peri-urban | Hypertension awareness, hypertension treatment, |  | ***** |
|  |  |  |  | Focus group discussion, Key | Uncontrolled hypertensive persons of age 18-70 years | 20 (10/10) |  |  |  |  |  |  |
| **3** | Dhimal et al. (2020) | Cross-sectional, quantitative study | Multistage cluster sampling | Structured interview | Initially sampled 6328 participants of age 15-69 years. Among them, 296 hypertensive participants who were prescribed antihypertensive medication by health workers, but stopped taking antihypertensive medications at least from last 12 days | 296 (120/176) | February to May 2019 | Nationwide | - | Adherence to medication | Nationally representative sample for assessing the non-communicable disease risk factors. This study was not specifically designed for assessing barriers to treatment and control of hypertension | *** |
| **4** | Khan et al. (2013) | Cross-sectional, quantitative study | Not clear | Structured interview | Treated hypertensive persons | 79 (44/35) | 2012 | Pokhara | Urban | Hypertension control | No clear explanation on sampling procedures, no adjustment of confounding in analysis | ** |
| **5** | Khanal et al. (2017) | Cross-sectional, qualitative study | Purposive sampling | In-depth interview | Health care providers (health assistants, pharmacy assistants, nurse, specialised nurse, practising pharmacists, chief hospital pharmacist, doctors, and specialised doctor) | 9 (4/5) | March 2015 | Lekhnath Municipality, Kaski | Not specified | Hypertension treatment | Small sample size, participants are not the hypertensive patients | ** |
| **6** | Maharjan S (2016) | Cross-sectional, quantitative study | Purposive sampling | Structured interview | Hypertensive patients of age 18 years and above and who were on treatment | 85 (53/32) | January and March 2016 | Sahid Gangalal National Heart Center | Urban | Treatment adherence | No adjustment of confounding in analysis | ** |
| **7** | Oli et al. (2014) | Cross-sectional, qualitative study | Non-probability sampling | In-depth interviews | Patients with confirmed hypertension, diabetes, heart disease for at least one year, Age 20 years and older | 13 (6/7) | May and July 2013 | Bhaktapur | Peri-urban | Hypertension treatment | Participants belonged to non-hypertensive patient as well | ***** |
| **8** | Roka et al. (2020) | Cross-sectional, qualitative study | Sequential sampling | Structured interview | Hypertensive participants attending an outpatient clinic at the study centre | 216 (110/106) | July to August 2017 | Shree Birendra hospital | Not specified | Treatment adherence | Non-probability sampling | *** |
| **9** | Shrestha et al. (2018) | Cross-sectional, qualitative study | Random sampling | Focus group discussion | Newly diagnosed hypertensive patients of age 18 years and above | 35 (26/9) | Dec 2015 to Feb 2016 | Dhulikhel Municipality | Peri-urban | hypertension awareness, hypertension treatment, hypertension control | Non-response rate is 27.8 | **** |
| **10** | Simkhada R (2012) | Cross-sectional, quantitative study | Random sampling | Structured interview, clinical measurements | Hypertensive participants diagnosed and under medication at least from last 6 months | 147 (83/64) | 1 Feb 2011- 31 July 2011 | Bir Hospital, Kathmandu, Nepal | Not specified | Hypertension control | Small sample size; no age and sex-adjusted analysis | ** |
