# Supplementary material for: Barriers, Enablers and Strategies for the Treatment and Control of Hypertension in Nepal: A Systematic Review
Source: Front Cardiovasc Med. 2021 Oct 11;8:716080. doi: 10.3389/fcvm.2021.716080 (PMC8542767; doi:10.3389/fcvm.2021.716080)
Supplement: Supplementary file 7 [file Table_7.docx]

**Table 7. Characteristics of the studies on the effectiveness of strategies to improve hypertension treatment and control in Nepal**

| **Sn** | **Study ID** | **Study types** | **Study date and sites** | **Participants** | **Participant recruitment** | **Intervention** | **Sample size total (treatment/control)** | **Outcome** | **MMAT score** |
| --- | --- | --- | --- | --- | --- | --- | --- | --- | --- |
| **1** | Humagain et al. (2015) | Prospective comparative study | January 2015 to April 2015; Dhulikhel Hospital, Kathmandu University Nepal | Hypertensive patients of 40-80 years of age attending cardiology OPD | Enrolled all the hypertensive patients who attended the study site during the study period and divided them into three groups based on the three antihypertensive medication they used: hydrochlorothiazide 25 mg or amlodipine 5 mg or enalapril 5 mg | Participants received an antihypertensive medication (any of hydrochlorothiazide 25 mg or Amlodipine 5 mg or Enalapril 5 mg) and health education. | 172 (NA/NA) | Systolic blood pressure  Diastolic blood pressure | *** |
| **2** | Khadka et al. (2010) | Randomised controlled trial | 2000-2002; Yoga and lifestyle clinics, Department of Physiology, BPKIHS | Essential hypertensive patients of 30-60 years of age, without diabetes | Purposively selected the participants and Systematically randomised them into yoga and control group | Treatment group participants underwent for yoga practice scheduled for half an hour at least for 6 days in a week for 6 weeks; yogic practices were strengthening exercise (5 min), yogic asanas (6-7 minutes), Shavasan (5 minutes), meditation (5 minutes), pranayam (4 minutes) | 14 (7/7) | Systolic blood pressure  Diastolic blood pressure | *** |
| **3** | Kumar et al. (2019) | Uncontrolled Before and After study | 1 December 2016–31 May 2018; Achham, Nepal | Hypertensive patients of age 18 years and above | Among the 488 hypertensive patients enrolled in the NCD program, the study selected 86% (340 out of 488) patients (all) with at least two follow up visits and having at least 12 week interval between baseline and endline | Intervention includes NCD management capacity development for healthcare workers, establishment of digital and electronic health records in the centre and individual (patient) level counselling for preventive and treatment of NCDs. | 340 (340/-) | Hypertension control | *** |
| **4** | Neupane et al. (2018) | Clustered randomised trial | 2015-2016; Lekhanath municipality, Kaski | General population (normotensive, pre-hypertensive, hypertensive) of 25-65 years of age | Participants were selected from 14 clusters. The municipality was divided into 15 clusters out of which 14 clustered were randomised into intervention and control group; 1:1 ratio. | The treatment group participants received health education on major NCD risk factors, had their blood pressure measurement and were referred (only for hypertensive participants) to health centre by the trained Female Community Health Volunteer in every four months/year. | 1638 (939/699) | Systolic blood pressure Diastolic blood pressure | **** |
| 5 | Sharma et al. (2014) | Uncontrolled Before and After study | August 2012 to April 2013; Sankalpa Pharmacy, Kaski, Nepal | Hypertensive patients of 31 years and above attending the pharmacist run hypertension clinic | Enrolled all the eligible hypertensive patients who attended the study site | Healthcare workers (pharmacist) led one- to one counselling - contained three counselling session at 0 date, two months and four months. The contents of counselling were lifestyle modification, treatment, and control of hypertension | 50 (50/-) | Systolic blood pressure  Diastolic blood pressure | *** |
