# Supplementary material for: Barriers, Enablers and Strategies for the Treatment and Control of Hypertension in Nepal: A Systematic Review
Source: Front Cardiovasc Med. 2021 Oct 11;8:716080. doi: 10.3389/fcvm.2021.716080 (PMC8542767; doi:10.3389/fcvm.2021.716080)
Supplement: Supplementary file 8 [file Table_8.docx]

**Table 8. Barriers and enablers of hypertension treatment and control reported in qualitative studies**

| **Domain** | **Barriers** | **Enablers** |
| --- | --- | --- |
| Health system-related (excluding provider and patient) | Unaffordable services [1]; Lack of human resources and diagnostic tools [2] | Free essential medicines at health centre [2] |
| Health care provider-related | Communication gap between patients and providers regarding medication use and follow-up visits [1-4]; Inadequate counselling on lifestyle modifications [1-4]; Long waiting hours [1, 3]; Lack of national guidelines for hypertension treatment [3]; Provider's unsupportive behaviours [1] | - |
| Patient-related | Non-adherence [1, 3]; Irregular follow-up visits [2, 3]; Poor help-seeking behaviours [2]; Reluctance to change behaviours [1]; Reluctance to take medication [1].; Perceived side effects of anti-hypertensive medication [1, 3]; Self-medication [2]; Lack of family support [1]; Financial hardship [4] | Family support [1, 4]; Perceived seriousness of the illness [1]; Self-motivation [1]; Use of reminders for medication [1]; Use of medication containers [1] |

1. Shrestha, S., et al., *Barriers and facilitators to treatment among patients with newly diagnosed hypertension in Nepal.* Heart Asia, 2018. **10**(2).

2. Khanal, S., et al., *Use of healthcare services by patients with non-communicable diseases in Nepal: A qualitative study with healthcare providers.* Journal of Clinical and Diagnostic Research, 2017. **11**(6): p. LC01-LC05.

3. Devkota, S., et al., *Barriers to Treatment and Control of Hypertension among Hypertensive Participants: A Community-Based Cross-sectional Mixed Method Study in Municipalities of Kathmandu, Nepal.* Front Cardiovasc Med, 2016. **3**: p. 26.

4. Oli, N., et al., *Experiences and perceptions about cause and prevention of cardiovascular disease among people with cardiometabolic conditions: findings of in-depth interviews from a peri-urban Nepalese community.* Global health action, 2014. **7**(1): p. 24023-24023.
