# Supplementary material for: Barriers, Enablers and Strategies for the Treatment and Control of Hypertension in Nepal: A Systematic Review
Source: Front Cardiovasc Med. 2021 Oct 11;8:716080. doi: 10.3389/fcvm.2021.716080 (PMC8542767; doi:10.3389/fcvm.2021.716080)
Supplement: Supplementary file 9 [file Table_9.docx]

**Table 9. Barriers to hypertension treatment and control in quantitative studies**

| Domain | Barriers | variables studied | Effect on outcome variables | | Voting | Study quality |
| --- | --- | --- | --- | --- | --- | --- |
|  |  |  | Treatment non-adherence | Uncontrolled hypertension |  |  |
| Health system related | High cost of medicine | Expensive (vs. affordable) medicine [1] | Odds ratio = 5.2; 95% CI: 1.1-23.9 | - | ++ | Good |
|  |  | Expensive medicine [2] | 5% of those who did not take medicine |  |  | Fair |
|  | Unavailability of medicine | Medicine is not available [2] | 1.7% of those did not take medicine |  | + | Fair |
| Provider related | Long waiting time at hospital for appointment | long waiting hours (>20 minutes vs. ≤ 20 minutes) [3] |  | Odds ratio = 2.7; 95% CI: 1.1-6.8; | + | Fair |
|  | Lack of behavior change counselling from the health providers | No counselling [3] |  | Odds ratio = 2.6, 95% CI 1.2-6.4 | + | Fair |
|  | Multiple pills prescription | Multi-pills (more than one vs. one antihypertensive drug prescription) [1] | Odds ratio = 6.4; 95% CI: 1.2-33.4 |  | + | Good |
| Patient related | Lack of follow-up consultations | Lost to follow up (irregular vs regular) [1] | Odds ratio = 6.4; 95% CI: 1.2-33.4 |  | ++ | Good |
|  |  | Lost to follow up (no follow up vs regular follow-up) [3] |  | Odds ratio = 3.3, 95% CI: 1.2-9.1 |  | Fair |
|  |  | lack of blood pressure monitoring [4] |  | Odds ratio = 2.9, 95% CI: 1.0-8.0 |  | Fair |
|  | Poor adherence to medication | Non-adherence to medication (low adherence vs high adherence) [3] |  | Odds ratio = 9.1, 95% CI: 3.5-24.1; | ++ | Fair |
|  |  | Non-adherence to medication [4] |  | Odds ratio = 5.8, 95% CI: 2.7-12.6; |  | Fair |
|  | Lack of awareness on blood pressure target | Lack of awareness on blood pressure target [3] |  | Odds ratio = 3.2; 95% CI: 1.3-8.2; | ++ | Fair |
|  |  | Not aware of normal blood pressure [4] |  | Odds ratio = 2.8, 95% CI: 1.3-6.1; |  | Fair |
|  | Lack of awareness on blood pressure complications | lack of awareness on high blood pressure complications [4] |  | Odds ratio = 2.6, 95% CI: 1.2-6.1; | + | Fair |
|  | Comorbidity | Comorbidity (yes vs no) [5] | Odds ratio = 2.5; 95% CI: 1.3-4.9 |  | + | Fair |
|  | Others | Do not think drug is necessary [2] | 37.8% of those who did not take medicine |  | + | Fair |
|  |  | Perceived side effect of drugs [2] | 6.1% of those who did not take medicine |  | + | Fair |

1. Bhandari B, Bhattarai M, Bhandari M, Ghimire A, Pokharel PK, Morisky DE: **Adherence to Antihypertensive Medications: Population Based Follow up in Eastern Nepal**. *Journal of Nepal Health Research Council* 2015, **13**(29):38-42.

2. Dhimal M, Bista B, Bhattarai S, Dixit LP, Md Khursid Alam Hyder, Agrawal N, Rani M, Jha AK: **Repot on noncommunicable disease risk factors: STEPS survey Nepal 2019**. In*.*, vol. 1. Kathmandu, Nepal: Nepal Health Research Council; 2020: 394.

3. Devkota S, Dhungana RR, Pandey AR, Bista B, Panthi S, Thakur KK, Gajurel RM: **Barriers to Treatment and Control of Hypertension among Hypertensive Participants: A Community-Based Cross-sectional Mixed Method Study in Municipalities of Kathmandu, Nepal**. *Front Cardiovasc Med* 2016, **3**:26.

4. Simkhada R: **Study on blood pressure control status and predictors of uncontrolled blood pressure among hypertensive patients under medication**. *Nepal Medical College journal : NMCJ* 2012, **14**(1):56-59.

5. Roka T, Ghimire M: **Medication Adherence among Hypertensive Patients Attending a Tertiary Care Hospital in Nepal**. *Journal of Nepal Health Research Council* 2020, **17**(4):521-527.
